# Novel Tools in Determining the Physiological Demands and Nutritional Practices of Ontario FireRangers during Fire Deployments

**DOI:** 10.1371/journal.pone.0169390

**Published:** 2017-01-20

**Authors:** A. H. Robertson, C. Larivière, C. R. Leduc, Z. McGillis, T. Eger, A. Godwin, M. Larivière, S. C. Dorman

**Affiliations:** 1 Centre for Research in Occupational Safety and Health (CROSH)–Laurentian University, Sudbury, Canada; 2 School of Human Kinetics, Laurentian University, Sudbury, Canada; Oklahoma State University, UNITED STATES

## Abstract

**Introduction:**

The seasonal profession of wildland fire fighting in Canada requires individuals to work in harsh environmental conditions that are physically demanding. The purpose of this study was to use novel technologies to evaluate the physiological demands and nutritional practices of Canadian FireRangers during fire deployments.

**Methods:**

Participants (n = 21) from a northern Ontario Fire Base volunteered for this study and data collection occurred during the 2014 fire season and included Initial Attack (IA), Project Fire (P), and Fire Base (B) deployments. Deployment-specific energy demands and physiological responses were measured using heart-rate variability (HRV) monitoring devices (Zephyr BioHarness3 units). Food consumption behaviour and nutrient quantity and quality were captured using audio-video food logs on iPod Touches and analyzed by NutriBase Pro 11 software.

**Results:**

Insufficient kilocalories were consumed relative to expenditure for all deployment types. Average daily kilocalories consumed: IA: 3758 (80% consumption rate); P: 2945±888.8; B: 2433±570.8. Average daily kilocalorie expenditure: IA: 4538±106.3; P: 4012±1164.8; B: 2842±649.9. The Average Macronutrient Distribution Range (AMDR) for protein was acceptable: 22–25% (across deployment types). Whereas the AMDR for fat and carbohydrates were high: 40–50%; and low: 27–37% respectively, across deployment types.

**Conclusions:**

This study is the first to use the described methodology to simultaneously evaluate energy expenditures and nutritional practices in an occupational setting. The results support the use of HRV monitoring and video-food capture, in occupational field settings, to assess job demands. FireRangers expended the most energy during IA, and the least during B deployments. These results indicate the need to develop strategies centered on maintaining physical fitness and improving food practices.

## Introduction

Wildland fire fighters, called FireRangers in Canada, are exposed to a multitude of hazards, including: unpredictable fire behaviour and weather, unstable and rough terrain, heat/smoke, all while transporting and operating heavy equipment [1,2]. Prolonged physical exertion and negative energy balance, related to inadequate nutrition [3,4], may cumulatively impact FireRangers. Therefore, it is important that FireRangers be both physically fit and adequately fuelled.

Previous research globally [5,6] has highlighted the intense physical demands of this profession. Energy expenditure has been estimated at ~4500kcal/day [5,6]. This is comparable to military personnel during combat training [7], and athletes during training and competition [8,9,10]; suggesting that FireRangers are Occupational Athletes and should adopt structured fitness practices similar to these professional groups.

Given the observed physical demands, the need for adequate intake of quality nutrients is implied, yet FireRangers have previously been found to operate with a negative energy balance of ~500kcal/day [6]. Montain and colleagues (2008) also showed that FireRangers consumed ~3000kcal/day [11], which is below the estimated kilocalorie need reported previously [6]. A negative energy balance, over time, can substantially impact physical performance [3,12,13,14], leading to weight loss and muscle wasting, as well as decreased: strength, immune function, energy levels, and alertness [15].

In addition to the need for sufficient kilocalorie consumption, appropriate amounts of macro- and micronutrients are required. Canadian recommendations regarding Average Macronutrient Distribution Ranges (AMDR) for the general population indicate that 45–65% of kilocalories should come from carbohydrates, 10–35% from protein, and 20–35% from fat [3,16].

With respect to macronutrients, Ruby and colleagues [6] reported FireRanger diets consisting of 53±6.9% carbohydrate, 14±2.4% protein, and 32±6.2% fat. Likewise, Cuddy and colleagues [17] found FireRangers consumed 59±6% carbohydrate, 12±3% protein, and 31±7% fat during the 12hr shifts in which they collected data. Both of these studies indicate that FireRanger’s AMDRs meet the Canadian Food Guide recommendations [3,16]. However, the Canadian Food Guidelines were developed for the general population, which does not adequately describe this workforce. In fact, athletes commonly augment their protein intake to promote muscle and tissue recovery and there is evidence to support this practice [3,18]. Likewise, exercise physiologists promote the enhanced consumption of carbohydrates for optimal performance during high-intensity and prolonged activities. Specifically, carbohydrates are essential to replenish muscle glycogen stores [3,17,18,19,20,21,22], blunt inappropriate inflammatory responses from activity, reduce the risk of infection and promote muscle recovery and repair [23,24,25]. In addition, carbohydrates are required for the production of key hormones involved in muscle building, including testosterone and growth hormone [26].

Micronutrient consumption profiles of FireRangers have not been assessed to date; however, micronutrients are known to be important for optimal physical performance [3,18,27] and overall wellbeing [3,28].

In Canada, fire suppression activities of FireRangers can be divided into three categories: Initial attack (IA), Project Fire (P), and Fire Base (B) deployments. IA deployments involve first-response, fire-suppression duties; physical activity is intense and duration of deployment is typically short, but varies according to fire intensity. IA deployments are in remote, unsettled locations and the primary objective is to contain fires; these deployments are anecdotally perceived as being ‘the most stressful’. P fires are the longer-term efforts of fire-suppression. They involve prolonged, moderately-intense physical activity, with duties related to clean up and smoulder extinction. B activities are those that are performed when FireRangers are not on fire deployment but are reporting daily to the Fire Management Headquarters (FMH). Although job demands for Canadian FireRangers are similar to FireRangers globally [29,30,31], data specific to the Canadian fire suppression is lacking.

To date no studies have: i) examined energy expenditure and food consumption *simulataneously* amongst Canadian FireRangers; ii) considered these variables under varying deployment conditions; nor iii) examined the macro- and micronutrient consumption patterns of these workers.

A key reason for this is the laborious nature of the data collection specific to energy expenditure and food consumption in the occupational setting. Past methodologies for energy expenditure were either only possible in the laboratory setting or, as is the case for doubly-labelled water, require regular, on-site personal sampling [29,32,33], which is not feasible during remote fire deployments. Likewise, food consumption analysis has classically relied on either recall or diary-keeping, both of which are notoriously faulty [34,35,36,37].

In contrast, heart rate variability (HRV) monitors can demonstrate moment-to-moment energy expenditure as estimated by the surrogate measure of heart rate rhythm[38,39,40]. The accuracy of HRV-based energy expenditure estimates has been validated against indirect calorimetry in the laboratory setting [38,39,40]. In addition, based on the relationship between HRV and autonomic nervous system activity, HRV data can also provide information about stress and recovery, as defined by Firstbeat Technologies [41]; these devices have yet to be used in a wildland fire fighting setting.

Likewise, in the field of nutrition, the use of innovative technologies has also been emerging in the literature as researchers try to improve dietary assessment methodologies. Mobile phone, scan and sensor-based technologies have improved real-time recording at eating events, and are generally thought to enhance the data collection process, (i.e. less laborious), while being as-accurate-as, and preferred-to, journal-recording of food consumption [34].

In summary, the literature highlights the need to perform a more detailed assessment of the energy demands and nutritional habits specific to Canadian FireRangers, to develop strategies for their unique working environment; and novel technology now exists to do so effectively. Therefore, the purpose of this study was to: i) simultaneously quantify daily energy expenditure and food consumption in FireRangers; ii) quantify energy demands and physiological responses of FireRangers based on deployment type; and iii) assess the nutritional quality of foods consumed by FireRangers, all, using two novel technologies: HRV-monitoring and audio-video journaling.

## Methods

### Participants

During the 2014 fire season, 23 FireRangers were recruited to participate in this study. Two participants withdrew from the study: one sustained an injury and was therefore absent from the workplace; and one voluntarily withdrew for personal reasons, resulting in a final sample of twenty-one participants (n = 21). All participants provided written, informed consent prior to the start of data collection, and this study received ethics approval from the Laurentian University Research Ethics Board. All participants (n = 21) were male with a mean age of 29.8 ± 8.5 years, height of 180.6 ± 8.1cm, weight of 85.3 ± 11.8kg, and activity class of 7.0 ± 0.6 (self-reported according to Firstbeat activity class categories) [42].

### Study Design

To recruit participants for the study, members of the research team met prospective participants (18 crews x 4 FireRangers = 72 potential participants) on two occasions prior to the start of the 2014 fire season. Upon receiving informed consent (n = 23; 32% response rate), the research team met with the participants to collect baseline information (age, height, weight, Heart Rate (HR_rest_, HR_max_ (HR_max_ = 208–0.7age)) [43], activity level [42], and provided individual training to the participants on the data collection protocols as well as operation and maintenance of the data collection equipment. As a support resource, instructional videos and images detailing all aspects of the aforementioned training were loaded onto iPod Touches provided to each participant. Participants were also provided with researcher contact information if further assistance was required, as well as for in-season correspondence. Participating FireRangers were asked to carry several small pieces of data logging equipment with them throughout the season: a BioHarness3 HRV monitor (Medtronic–Annapolis, MD), an iPod Touch (Apple–Cupertino, CA), an ActiSleep monitor (ActiGraph–Pensacola, FL), and a compact battery to charge the devices as necessary (Anker Astro Pro2 20000mAh Multi-Voltage External Battery). Participants were instructed to carry and utilize the equipment during all deployment types throughout the fire season and use them until they returned from each deployment, at which time the data would be downloaded and the devices returned to participants prior to their next deployment. FireRangers were not assigned specific days on which to collect data given that fire activity is unpredictable. Instead, FireRangers were provided with an individual equipment package and were responsible for initiating use of the equipment upon notice of fire deployment. FireRangers were also asked to collect data during extended periods in which they were aware they would be spending their shifts solely at the fire base. During deployments, participants were instructed to initialize and wear their BioHarness3 module upon waking in the morning until going to sleep at night, at which time they were required to charge the device overnight. From sleep onset until waking, participants were instructed to wear the ActiSleep monitor. These latter devices were not worn during the day due to concerns regarding exposure to water and submersion that would exceed their functional capacity. Additionally, during each day of data collection, participants were instructed to use the video recording function of the iPod Touch to document all food consumed.

### Heart Rate Variability (HRV) Monitoring

HRV monitoring was used to estimate the energy expenditure of FireRangers during deployments. BioHarness3 units from Zephyr Technologies were selected as they were specifically designed for use in athletes and emergency responders [44]. HRV measures are also representative of autonomic nervous system activity (i.e. parasympathetic and sympathetic activity), therefore HRV analysis was used to characterize the demands of FireRangers’ varied working conditions with corresponding physiological reactions (i.e. physically active, physiological stress, and physiological recovery) simultaneously [38,41]. HRV data was analyzed in Firstbeat SPORT software (Firstbeat Technologies–Jyväskylä, Finland), which defines:

- **physical activity—**as periods in which an individual exceeds 30% of their HR-derived VO_2max_;
- **stress—**as periods in which sympathetic nervous activity is dominant in relation to parasympathetic nervous activity; and
- **recovery**—as periods in which parasympathetic nervous activity is dominant in relation to sympathetic nervous activity [41].

Additionally, periods not categorized as any of the aforementioned states is defined as “other” [41]. Using this categorization, the Firstbeat SPORT software produces reports for each day logged by participants, representing each category as a percentage of total device wear time.

Each participant was provided with an individually calibrated (age, height, weight, HR_rest_, HR_max_) BioHarness3 module and chest strap, as well as a charging cradle and portable battery pack. Participants wore the monitors during waking hours and removed the unit for charging when going to sleep each day.

### Sleep Monitoring

Total sleep time (TST) was measured using ActiSleep monitors from ActiGraph. This wrist-worn device uses accelerometry to measure TST, has been validated against the gold-standard of polysomnography, and been deemed ideal for use in field studies with healthy individuals [31,45,46,47]. Average FireRanger TST was used for the purpose of determining 24hr energy expenditure.

### Food Logs

During Base Camp and Project deployments, each FireRanger was provided an iPod Touch to estimate energy intake (EI) and nutritional quality data, through use of the audio-video recording function. FireRangers were instructed to film the contents of their meals while simultaneously dictating portion sizes and ingredients that are unapparent from the visual alone (e.g. coffee in portable container). This method is a novel adaptation of the photographic food-logging method that has been used successfully in previous research [48,49] and has been deemed to be a more accurate and reliable method than self-report methods (i.e. recall) [34,35,36,37]. FireRangers were unable to self-record food consumption during IA, due to the nature of this type of deployment; therefore IA food data were examined differently. IA deployments are only two, consecutive days; during this time, pre-packaged, frozen and dry foods are dropped with the FireRangers and are the *only* foods available to them until supply runs can reach their location. Therefore, researchers photographed all contents of the IA foods provided to FireRangers and, assuming FireRangers divided the foods equally amongst their crew, subsequently determined individual FireRanger daily food availability.

### HRV Data Analysis

The raw HRV (R-R interval) data extracted from the BioHarness3 units were analyzed using Firstbeat SPORT software. Individual profiles were created using participant baseline information (age, height, weight, HR_rest_, HR_max_, activity class) according to software calibration requirements [40,42].

### Nutrition Data Analysis

The contents of the P and B food logs and the contents of IA food photos were inputted into NutriBase Pro 11.0 nutritional analysis software (CyberSoft–Phoenix, AZ) allowing for detailed energy intake, as well as macro- and micronutrient analysis.

The results of the food log analyses were then compared to established energy intake, macro- and micronutrient recommendations [16,50,51]. Since it is recommended that a minimum of three, consecutive days of food data is required to adequately represent food consumption patterns [52,53], only deployments with a minimum of three consecutive days were analysed for macro- and micronutrient content for each type of deployment.

### Daily Energy Expenditure

Given the need to charge the BioHarness3 units nightly, 24hr HRV monitoring was not possible. Upon downloading the data from the devices, it was observed that participants did not wear the BioHarness3 continuously from the time they woke up until they went to bed, as requested (See Table 1). Therefore, 24hr Energy Expenditure (EE) was estimated using: individual, average, and hourly EE observed for each deployment type; average sleep time (obtained from ActiSleep data); and MET values from the Compendium of Physical Activities [54]. Based on the working time guidelines set for FireRangers, the maximum of 16hrs/day was multiplied by EE_1hr_ for each type of fire deployment (IA and P); a) This provides an estimation of the maximum daily energy expenditure specific to each deployment type relative to the intensity observed during the 2014 fire season; b) Estimating daily energy expenditure using a shorter shift length (e.g. 50% of maximum daily working hours– 12hrs/day) would mean having to infer participant activities for the remaining hours, decreasing the ability of the estimates to reasonably represent each deployment type. For B deployments 12hrs/day was used for daily EE estimation, as this is the length of shift FireRangers work during days spent at headquarters. A total of 12 participants wore the ActiSleep monitor on 152 nights throughout the 2014 fire season, and average TST regardless of deployment type was found to be 364 ± 61.2min/night (~6 ± 1hrs/night). This finding is consistent with the TST observed in Australian Rangers during deployments [55,56]. Accordingly, 6hrs/night was used for daily energy expenditure estimation by multiplying this value by the corresponding Compendium of Physical Activities value for sleeping (1.0METs) [54]. For IA and P deployments, the remaining 2hrs, and 6hrs for B deployments, were conservatively estimated using the MET value for sitting/standing quietly (1.3METs) [54] as it cannot be assumed what activities FireRangers may perform during this time. The 24hr EE estimation formulas for each deployment type are listed below.

$$\begin{array}{l} Initial\;Attack–EE_{IA24est}=\left(16*EE_{1hrIA}\right)+\left[2*\left(1.3METs*kg\right)\right]+\left[6*\left(1METs*kg\right)\right]\\ Project\;Fire–EE_{P24est}=\left(16*EE_{1hrP}\right)+\left[2*\left(1.3METs*kg\right)\right]+\left[6*\left(1METs*kg\right)\right]\\ Fire\;Base–EE_{B24est}=\left(12{*}_{EE1hrB}\right)+\left[6*\left(1.3METs*kg\right)\right]+\left[6*\left(1METs*kg\right)\right] \end{array}$$

**Table 1:** Deployment-specific results.

	Deployment Type		
	Initial Attack (IA)	Project Fire (P)	Fire Base (B)
Total participants (HRV Data)	15	10	13
Total HRV shift logs (days)	37	36	35
Mean shifts logged per participant (days ± SD)	2.6 ± 1.1	3.6 ± 1.4	2.7 ± 1.6
Mean BioHarness3 log time (hours ± SD)	8.9 ± 3.7	11.5 ± 2.5	10.6 ± 2.4
Mean hourly energy expenditure (kcal/hr ± SD)	250 ± 61.9	202 ± 79.4	137 ± 51.1
Mean peak energy expenditure	a) 16 ± 2.6	a) 14 ± 4.9	a) 11 ± 4.1
	b) 11.3 ± 1.6	b) 9.9 ± 3.2	b) 8.1 ± 3.4
a) (kcal/min ± SD)			
b) (METs ± SD)			
Mean daily energy expenditure (kcal/day ± SD)	4538 ± 1006.3	4021 ± 1164.8	2842 ± 649.9
Mean daily energy intake (kcal/day ± SD)	• 100% = 4698	2945 ± 888.8	70.8
	• 80%* = 3758		
Mean daily energy balance (kcal/day ± SD)	• 100% = 160 ± 1006.3	-1063 ± 1499.0	-409 ± 851.9
	• 80%* = -780 ± 1006.3		
Mean physiological response profile (% total log time)	• 22.5 ± 10.3%—Physical Activity	• 16.4 ± 11.7%—Physical Activity	• 8.3 ± 6.0%—Physical Activity
	• 15.8 ± 13.9%—Stress 14.4 ± 18.9%—Recovery	• 4.6 ± 6.0%—Stress 32.3 ± 23.4%—Recovery	• 8.6 ± 14.6%—Stress 63.3 ± 23.5%—Recovery
	• 47.3 ± 16.6%—Other	• 46.7 ± 24.3%—Other	• 19.8 ± 14.0%—Other

* Previous research regarding food consumption patterns in wildland fire fighters indicated an 80% consumption rate of provided rations [6].

### Statistical Analysis

All statistical analyses were performed using IBM SPSS 20 (Armonk, New York). Results are shown using the mean ± SD. One-way ANOVA’s were used to detect significant differences between deployment-specific energy expenditure, physiological reactions, and energy balance. Independent t-tests were used to detect significant differences between energy intake and macronutrient/micronutrient profiles for P and B deployments; IA energy intake and macronutrient/micronutrient profiles were established from the available IA food supplies and therefore did not have a distribution of values that could be used in an ANOVA for comparison with the corresponding P and B values. Significance was accepted at the level of p<0.05.

## Results

### Deployment-Specific Energy Expenditure

**Fig 1:**
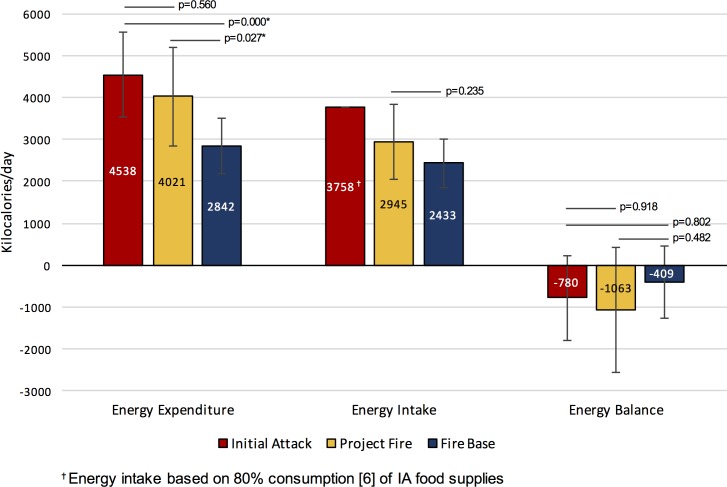
Deployment-specific daily energy expenditure, energy intake, and energy balance.

Table 1 summarizes deployment-specific descriptive statistics for the energy expenditure data including the number of shift logs where HRV data were collected and number of participants from whom the data were derived during initial attack (IA), project fire (P), and fire base (B) deployments. The hourly (EE_1hr_), peak (EE_peak_) and daily (EE_24hr_) energy expenditure (EE) data for each deployment type are also detailed in Table 1 and the daily EE data are further depicted in Fig 1.

Our main findings for EE are that hourly, peak and daily EE levels are typically lower for work conducted at the base compared to that conducted during the fire deployments (EE_1hrIA_> EE_1hrB_ p = 0.000; EE_1hrP_ > EE_1hrB_ p = 0.001; EE_peakIA_> EE_peakB_ p = 0.000; EE_peakP_> EE_peakB_ p = 0.013; EE_IA-24hr_> EE_B-24hr_ p = 0.001; EE_P-24hr_ > EE_B-24hr_ p = 0.027). Furthermore, hourly EE during IA deployments was higher compared to P fires (EE_1hrIA_ > EE_1hrP_ p = 0.014) (Table 1 and Fig 1).

### Deployment-Specific Energy Intake and Nutritional Content

**Fig 2:**
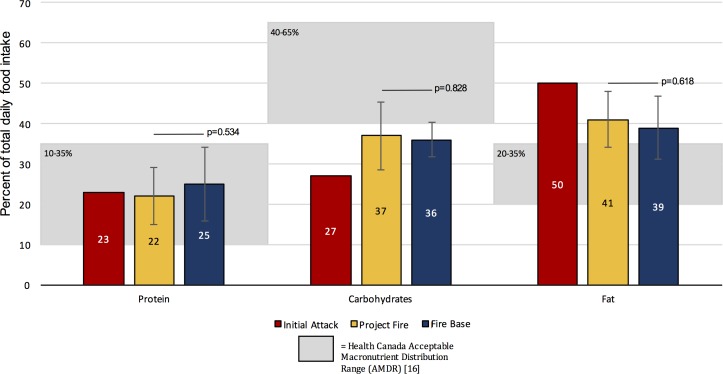
Deployment-specific macronutrient profiles.

Detailed energy intake, as well as macro- and micronutrient data for each deployment type are presented in Table 1, Table 2, Fig 1 and Fig 2. Since we were unable to collect data over three consecutive days for IA deployments, we report average intakes for this deployment type only for comparative purposes (Table 1, Fig 1). Statistical analyses of P fire and B data suggest that overall energy intake in kcal was similar between P fire and B deployments (Fig 1) and that energy intake in terms of %protein, %carbohydrates, and %fat and in terms of micronutrient content was similar (Table 2, Fig 2). We also note that protein intake generally falls within the recommended Health Canada Acceptable Macronutrient Distribution Range (AMDR), but that carbohydrate and fat intake fall respectively under and over the AMDR (Fig 2). As for micronutrient content, a lower-than-recommended consumption of fibre, potassium, vitamins D and E and a higher-than-recommended intake of sodium and iron were noted (Table 2).

**Table 2:** Deployment-specific food log descriptive statistics, macronutrient profiles, and micronutrient consumption relative to recommended dietary allowance (RDA) and adequate intake (AI) guidelines [36].

	Deployment Type		
	Initial Attack	Project Fire	Fire Base
Total participants (Food Logs)	NA**	5	7
Total food logs >3 Days*	NA**	6	7
Mean shifts logged (days ± SD)	NA**	4.2 ± 0.8	4.7 ± 1.6
Mean Macronutrient Profile (% total intake ± SD)	• 23%—Protein • 27%—Carbohydrates • 50%—Fat	• 22 ± 7.1%—Protein • 37 ± 8.4%—Carbohydrates • 41 ± 6.9%—Fat	• 25 ± 9.1%—Protein • 36 ± 4.3%—Carbohydrates • 39 ± 7.7%—Fat
Fiber (%AI)	68.4	80.7 ± 35.6	66.2 ± 49.4
Iron (%RDA)	347.5	270.8 ± 81.6	219.6 ± 97.3
Magnesium (%RDA)	50.1	87.5 ± 27.6	61.4 ± 49.6
Selenium (%RDA)	181.3	375.5 ± 179.3	231.4 ± 131.3
Zinc (%RDA)	151.8	150.0 ± 50.4	105.2 ± 36.3
Phosphorus (%RDA)	162.2	278.8 ± 143.7	179.6 ± 120.6
Calcium (%RDA)	135.9	93.9 ±35.2	82.1 ± 24.8
Sodium (%AI)	369.4	310.7 ± 142.6	279.9 ± 134.3
Potassium (%AI)	32.5	80.4 ± 26.6	53.4 ± 37.4
Vitamin A (%RDA)	96.0	246.3 ± 210.9	148.4 ± 195.4
Vitamin B12 (%RDA)	241.7	375.0 ± 320.6	333.3 ± 302.4
Vitamin C (%RDA)	34.4	146.3 ± 58.1	151.3 ± 259.3
Vitamin D (%RDA)	62.4	42.8 ± 52.9	16.9 ± 13.4
Vitamin E (%RDA)	58.5	116.1 ± 143.8	40.2 ± 33.1

* Food records must be kept for a minimum of 3 consecutive days to be used for analysis [52,53].

** Initial Attack (IA) deployments are classified as less than 48 hrs, therefore contents of pre-packaged IA food supplies to be sent on deployments were photographed and used for analysis.

### Deployment-Specific Energy Balance

In general, the energy balance for all deployment types was negative indicating that more energy was expended compared to energy intake (Fig 1) and this was true for all deployment types. When comparing the energy balance for IA (EB_IA_) using the 100% energy intake estimates (i.e. EB_IA-24hr_ 4698 kcal/day, Table 1), we noted a greater negative energy balance for EB_IA24hr_ compared to P fire EB_P-24hr_, (p = 0.038). Otherwise, no other differences were noted.

### Deployment-Specific HRV Activity Data

**Fig 3:**
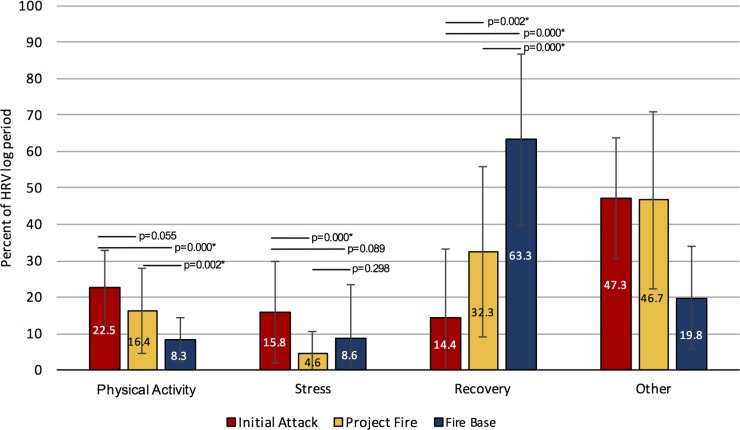
Deployment-specific percentage time spent in periods of physical activity, stress, and recovery.

The HRV data were categorized according to periods of either physical activity, physiological stress or physiological recovery. This analysis revealed that the total time engaged in physical activity during IA deployments was greater than that recorded at base (p = 0.001) and nearly higher than that observed for P deployments (p = 0.055). Furthermore, total time spent in a state of physiological stress during IA deployments was higher than P deployments (p = 0.001), but not greater than B deployments (p = 0.089). Finally, time spent in a state of physiological recovery was lowest during IA compared to P (p = 0.002) and B deployments (p = 0.001) whereas no differences were observed between P and B deployments (Table 1 and Fig 3).

## Discussion

This study employed two novel methodologies in an occupational field setting: HRV monitoring for quantifying and qualifying energy expenditure and physiological demands, as well as audio-video food diaries to quantify and qualify nutrient consumption. We found that these methodologies were well tolerated by participants and allowed us to successfully collect data regarding the physical activity and food consumption behaviours of FireRangers while they were working in remote locations. Using this data we conclude that Canadian FireRangers: i) should be considered ‘Occupational Athletes’ based on their physical activity levels; ii) are not consuming adequate kilocalories relative to the demands of their work activities during all fire deployment types, including an under-consumption of carbohydrates [3,16,18]; and iii) are not consuming appropriate amounts of some micronutrients, which may negatively affect their work performance and long term health. We recommend that resources for Canadian FireRangers be revised to include strategies to improve nutritional practices [3,18].

HRV monitoring has been previously shown to be a valid measure of energy expenditure estimation in the laboratory and during free-living conditions [38,39,40]. Our energy expenditure estimates using this method, reflect previously reported results, using doubly-labelled water [5,6].

Photographic dietary assessment, previously validated in the literature [34,35,36,37,48,49], was modified for use in this study. The audio-video recording of meals, allowed for the independent collection of food data during deployments, with both a photographic and verbal recording of meals, prior to dining, replacing previous methods of self-report (i.e. survey, diary, recall), which are largely considered to be inaccurate and unreliable [57,58,59]. Photographic diaries have been previously shown in the literature to be preferred by study participants over self-report methods [35], which is advantageous given established weaknesses of the latter [57,58,59]. The results from this study support these previous findings and support the use of this novel methodology for the collection of food data in daily living conditions.

The HRV data from this study confirmed that the fire suppression activities performed by Canadian FireRangers demand high energy expenditure [54]. Consistent with Rodriguez-Marroyo and colleagues (2011), our data shows that energy expenditure was highest during IA deployments and lowest at B [60]. To contextualize these energy expenditure values, the measures were converted to METs (kcal/kg/hr) for comparison with the Compendium of Physical Activities [54]. These comparisons indicated that at peak intensity, tasks performed are more intense than those during structural fire fighting (7-9METs), and comparable to competitive ice hockey and soccer (10METs), as well as cross-country running (9METs) [54]. The Compendium of Physical Activities underlines this finding by listing the task of inclined walking while carrying 50-74lbs as 10METs [54]; this specific task is regularly performed by FireRangers, who carry heavy equipment and supplies across rough terrain. Comparing FireRanger energy demands to the Compendium of physical activities provides a pragmatic way to contextualize the profession in terms of intensity, lending support to the notion that FireRangers are ‘occupational athletes’.

It is worth noting that the 2014 fire season in Ontario, when the data were collected, was one of the lowest intensity fire seasons recorded in recent years [61]. We would therefore anticipate that during an average intensity fire season, the hourly average and peak energy expenditures might be higher. This spectrum of observed, activity intensities that comprise wildland fire suppression duties should be considered when developing fitness programs and considering nutrient needs.

The audio-video food diaries provided valuable insight into the quality of diet and quantity of kilocalories consumed during deployments. Average food consumption during IA deployments was estimated at 3758kcal/day consumed (based on a calculated 80% of all foods supplied [11]). While averages during P and B deployments were found to be consistent with the daily intakes observed in American FireRangers (~3000kcal/day) reported by Montain and colleagues (2008) [11]. These levels are also consistent with the average daily energy intake of Canadian adults in the same age range (~3000kcal/day) [62] and are equivalent to Health Canada’s recommendations for *moderately* active adults [50]. Therefore, FireRangers are typically eating less kilocalories than they expend during all deployment types. Given that FireRangers perform tasks equivalent to the intensity of competitive sports (described above), kilocalorie enhancement in this population represents a key modifiable factor to optimize workplace performance and worker health and wellbeing.

The negative energy balances recorded during IA and P deployments, are important, due to the inherent risks associated with fire fighting and the need for adequate energy expenditure during an emergency. However, the negative energy balance at base camp is equally important, since it suggests that FireRangers are not ‘catching up’ from short-term deficiencies during recovery periods. Although these findings are consistent with previous studies amongst FireRangers [5,6,11] they are particularly concerning given that extended periods of negative energy balance can substantially impact physical performance [3,12,13,14], leading to weight loss and muscle wasting, as well as decreased: strength and performance, immune function, energy levels, and alertness, all of which can contribute to accident or injury occurrence [15]. Since energy balance is modifiable, it is advised that intervention strategies (i.e. workshops, eLearning modules etc.) be implemented to increase daily energy intake to match the energy demands for all deployment types.

In addition to the need for sufficient kilocalorie consumption to support activities, it is also important that workers consume appropriate amounts of macro- and micronutrients. Our results indicate that, regardless of deployment type, FireRangers exceeded daily fat intake and did not meet recommended carbohydrate intake, although protein intake was within an acceptable range [3,16]. These results deviate from the macronutrient distributions reported for American FireRangers by Ruby et al. (2002) [6] and Cuddy et al. (2007) [17] that were within Canadian macronutrient consumption guidelines [3,16]. FireRangers in the current study were also found to deviate from recommendations for athletes regarding daily macronutrient consumption relative to body weight (1.2–1.7g/kg protein and 6-10g/kg carbohydrates) [3]. FireRangers consumed 2.5g/kg, 1.9g/kg, and 1.8g/kg protein and 3.5g/kg, 3.4g/kg, and 2.8g/kg carbohydrates during IA, P, and B deployments respectively. Protein consumption up to 2.0g/kg is generally perceived to be harmless and may be beneficial for some athletes [3,30,51]. In fact, it is common for athletes to augment their protein intake to promote muscle and tissue recovery and there is evidence to support this practice [3,18]. Given that current protein consumption of our participants already meets and exceeds these recommendations, modifications are not recommended for this macronutrient.

In our opinion, the most significant finding in regards to the nutritional practices for FireRangers was inadequate carbohydrate consumption, which is approximately half of the recommended intake [3,18,30]. Carbohydrates are critical for high-intensity activities [22], which would be required in the case of an emergency situation; but are also essential for prolonged low-to-moderate intensity physical activity [25]. It is therefore imperative that FireRangers increase their carbohydrate consumption, in accordance with published guidelines, in order to provide the fuel necessary to achieve optimal and safe workplace performance particularly during demanding fire deployments.

With respect to fat consumption, given the overall under-consumption of total kilocalories and the fact that fat is presented as a percentage of total kilocalories, we would highlight diet changes that increased carbohydrates; if no further increases in fat consumption occur, total kilocalories from fat would fall within the upper- boundaries of acceptable range.

Micronutrient consumption profiles of FireRangers were also found to deviate from established recommendations [51] (Table 2), which may adversely affect their performance and health; acutely and chronically. The observed micronutrient consumption profile in the current study suggests, FireRangers may be at greater risk of decreased immune function [3,27,28], suboptimal performance [3,18], irregular bowel movements [30] and developing cardiovascular disease [30]. A simple solution to manage the extreme iron intake amongst this worker population would be an annual blood donation at the end of the fire season [63]. Vitamin D and Vitamin E consumption were noted to be below recommended values. Given that wildland firefighting is a summer occupation, adequate vitamin D is likely achieved via the skin. However, it is worth noting that FireRangers are also regularly exposed to particulate and gaseous elements from smoke that can induce oxidation, particularly in their airway [64]. Accordingly, inadequate Vitamin E consumption is concerning, particularly since this fat-soluble anti-oxidant could be beneficial for protecting cell membranes in the lining of the airways [65,66].

Deployment type largely impacts the eating habits of FireRangers. During the first two days of IA deployments FireRangers rely on pre-packaged dry and frozen food supplies that are prepared ahead of time and ready to be taken at a moments notice. The foods comprising the IA food supplies therefore consist of items that: are spoil resistant, easily transportable, quick and easy to prepare; all characteristics that are easily achieved with packaged, nutritionally sub-optimal foods. During P deployments, FireRangers typically consume communal meals for breakfast and dinner, while consuming a ration pack consisting of fruit items and various sandwiches (e.g. peanut butter and jelly, ham and cheese, chicken salad) between these meals. FireRanger eating habits during P deployments can therefore be considered a hybrid of both meal-based and snack-based food consumption, the latter of which is known to be more advantageous for occupational performance purposes [11,17]. Finally, when stationed at the Fire Base FireRangers are responsible for their own individual diets. Although this provides opportunity for normalization of food behaviours, educational programs aimed at teaching eating strategies to optimize recovery during this down time would be beneficial, given the data presented here.

In addition to providing energy estimates, the HRV data collected in this study was able to provide insight into the differing physiological reactions of FireRangers specific to each deployment type (Fig 3). Periods of physical activity were greatest during IA deployments further supporting the differences in activity intensity between direct and indirect attacks observed by Rodriguez-Marroyo and colleagues (2011) [60]. As expected, periods of higher physical activity were greatest during fire deployments compared to base work. While FireRangers are still required to perform physical labour while working at the base, in preparation for fire deployments, our findings indicate that these workers have an opportunity to rest and recover from the intense physical demands of fire suppression activities while they await subsequent fire deployments. Other research on Australian FireRangers has indicated that they are able to self-regulate their daily physical activity in order to maintain task performance and subjective measures of exertion over consecutive days performing fire suppression tasks [56,67]. This research indicates that FireRangers exhibit “activity synergy”. In other words, while still completing all work tasks at the same level of performance, more time is spent in periods of light physical activity during a shift, which results in higher intensity physical activity during the subsequent shift [67]. These findings are positive indicators of a FireRangers’ abilities to cope with the consistent physical demands of their occupation.

Stress as measured using HRV analysis in this study, was representative of increased sympathetic nervous system activity [41], and long-term sympathetic activation has been shown to lead to cardiac disease and health consequences [41,68,69]. Periods of physiological stress were lower during project fires relative to initial attacks and this is unsurprising when considering the nature of each deployment type. For instance, P fire deployments involve predictable activities of moderate intensity, whereas IA deployments are spontaneous and involve unpredictable fire behaviour and hazardous environmental conditions. Interestingly, levels of physiological stress were similar between project fire deployments and non-fire base work periods. The lack of consistent fire deployments and the low number of fires during the 2014 fire season may partially explain the stress reactions experienced by these workers.

Adequate recovery following intense physical activity is essential for FireRangers who may perform their duties for up to 14 consecutive days. Recovery as measured using HRV analysis in this study, is representative of increased parasympathetic nervous system activity [41]. In the current study, recovery periods were less pronounced during fire deployments particularly for IA compared to fire base work, where recovery periods were more substantial. This was expected given that FireRangers can work up to 16hr/day while deployed to a fire and undertake the highest intensity activities with reduced opportunities for recovery given the urgency inherent in this type of fire deployment. In this context, it is important that these workers have periodic recovery breaks throughout their shifts as this has been shown to be an effective fatigue mitigation and performance maintenance strategy [69].

## Limitations & Future Directions

While this study supports the use of novel technological applications, there are some limitations that should be addressed.

First, with respect to food data collection, a current limitation to the widespread application of food-analysis via video diaries is a gap in the technology capable of analysing the macro and micronutrient content. That is, researchers must still manually enter food items line-by-line. Therefore, although *data collection* can be streamlined, *data input* is still a highly laborious process. Software capable of automated food photo analysis and data entry would have a dramatic impact in the field of nutrition.

In addition, due in part, to the total number of HRV monitors and iPod touches available to the research team, the number of people from whom data could be collected from was limited. We recognize that our sample size was small and therefore that the data cannot be representative of all Canadian FireRangers.

## Conclusions

Adequate energy intake and proper nutritional practices are essential for maintaining performance levels and are therefore key components of optimizing workplace performance within the FireRanger occupation. This study supports the use of a modern technology, for the comprehensive evaluation of physiological variables and food consumption profiles, in free-living occupational settings, allowing for data collection in circumstances where a researcher’s presence is prohibited or obstructive.

## Supporting Information

S1 Data (XLSX)
